# Cox regression increases power to detect genotype-phenotype associations in genomic studies using the electronic health record

**DOI:** 10.1186/s12864-019-6192-1

**Published:** 2019-11-04

**Authors:** Jacob J. Hughey, Seth D. Rhoades, Darwin Y. Fu, Lisa Bastarache, Joshua C. Denny, Qingxia Chen

**Affiliations:** 1 Department of Biomedical Informatics, Vanderbilt University Medical Center, Nashville, TN USA; 2 Department of Biological Sciences, Vanderbilt University, Nashville, TN USA; 3 Department of Medicine, Vanderbilt University Medical Center, Nashville, TN USA; 4 Department of Biostatistics, Vanderbilt University Medical Center, Nashville, TN USA

**Keywords:** GWAS, Electronic health record, Time-to-event modeling, Cox regression

## Abstract

**Background:**

The growth of DNA biobanks linked to data from electronic health records (EHRs) has enabled the discovery of numerous associations between genomic variants and clinical phenotypes. Nonetheless, although clinical data are generally longitudinal, standard approaches for detecting genotype-phenotype associations in such linked data, notably logistic regression, do not naturally account for variation in the period of follow-up or the time at which an event occurs. Here we explored the advantages of quantifying associations using Cox proportional hazards regression, which can account for the age at which a patient first visited the healthcare system (left truncation) and the age at which a patient either last visited the healthcare system or acquired a particular phenotype (right censoring).

**Results:**

In comprehensive simulations, we found that, compared to logistic regression, Cox regression had greater power at equivalent Type I error. We then scanned for genotype-phenotype associations using logistic regression and Cox regression on 50 phenotypes derived from the EHRs of 49,792 genotyped individuals. Consistent with the findings from our simulations, Cox regression had approximately 10% greater relative sensitivity for detecting known associations from the NHGRI-EBI GWAS Catalog. In terms of effect sizes, the hazard ratios estimated by Cox regression were strongly correlated with the odds ratios estimated by logistic regression.

**Conclusions:**

As longitudinal health-related data continue to grow, Cox regression may improve our ability to identify the genetic basis for a wide range of human phenotypes.

## Background

The growth of DNA biobanks linked to data from electronic health records (EHRs) has enabled the discovery of numerous associations between genomic variants and clinical phenotypes [1]. Two salient characteristics of EHR data are the large number of correlated phenotypes and the longitudinal nature of observations. Although methods have recently been developed to handle the former [2, 3], approaches to make use of the latter in the context of genome-wide or phenome-wide association studies (GWAS or PheWAS) are less common. Cases are typically defined as individuals with evidence of a phenotype at any timepoint in their record, and most large-scale analyses to date have employed logistic or linear regression, which do not naturally account for the time at which a particular event occurs or the highly variable length of observation between patients.

Statistical modeling of time-to-event data has been well studied and frequently applied to the clinical domain [4]. One such method often used to identify genotype-phenotype associations is Cox (proportional hazards) regression [5]. Previous work has demonstrated the advantages of Cox regression over logistic regression for data having a small number of single-nucleotide polymorphisms (SNPs) or collected under particular study designs [6, 7]. To our knowledge, the extent to which these findings generalize to analyses of genome-wide, EHR-linked data remains unclear. Unlike most data analyzed by Cox regression, EHR data are collected for the purposes of clinical care and billing, and are only made available secondarily for research. Thus, not only may individuals leave the healthcare system prior to having an event (a common issue known as right censoring), but they enter the system at various ages (a phenomenon called left truncation).

Here we sought to compare the performance of Cox regression and logistic regression for identifying genotype-phenotype associations in genetic data linked to EHR data. Using both simulated and empirical data, we found that Cox regression shows a modest but consistent improvement in statistical power over logistic regression.

## Results

**Fig. 1:**
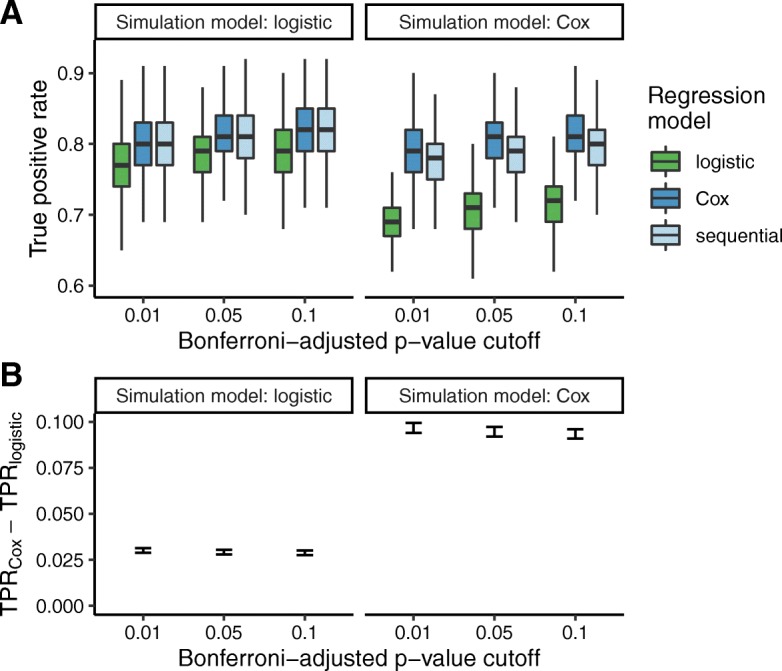
Comparing logistic regression and Cox regression on data simulated from either a logistic model or a Cox model (1000 simulations each). Each simulation included 100 risk alleles and 799,900 alleles not associated with the phenotype. True positive rate was calculated as the fraction of risk alleles having Bonferroni-adjusted *p*-value less than the given cutoff. **a** Boxplots of true positive rate for logistic regression, Cox regression, and the sequential strategy, across simulations from each simulation model. The sequential strategy used the p-value from Cox regression, if the unadjusted p-value from logistic regression was ≤10^− 4^. For ease of visualization, outliers are not shown. **b** 95% confidence intervals of the difference between the true positive rates of Cox and logistic regression

We first compared logistic regression and Cox regression based on their abilities to detect associations in data simulated from either a logistic model or a Cox model. In simulations from either model and at various *p*-value cutoffs, the true positive rate tended to be higher for Cox regression than for logistic regression (Fig. 1). As expected, the difference in true positive rates between the two regression methods was smaller when the data were simulated from a logistic model. In simulations from either model, both regression methods had mean false positive rates < 2·10^− 7^ even at the highest p-value cutoff. Based on our simulations, we would expect Cox regression to detect an additional 3 to 9 associations for every 100 true risk alleles, while falsely claiming 0.05 associations for every 10^6^ non-risk alleles.

Because Cox regression is less computationally efficient than logistic regression, previous work suggested a sequential strategy of running logistic regression on all SNPs, then running Cox regression on the SNPs that meet a particular logistic *p*-value cutoff [7]. The number of hypotheses and thus the threshold for Bonferroni correction do not change. In our simulations, this sequential strategy achieved a true positive rate similar to or slightly lower than Cox regression alone, and considerably higher than logistic regression alone (Fig. 1a).

**Fig. 2:**
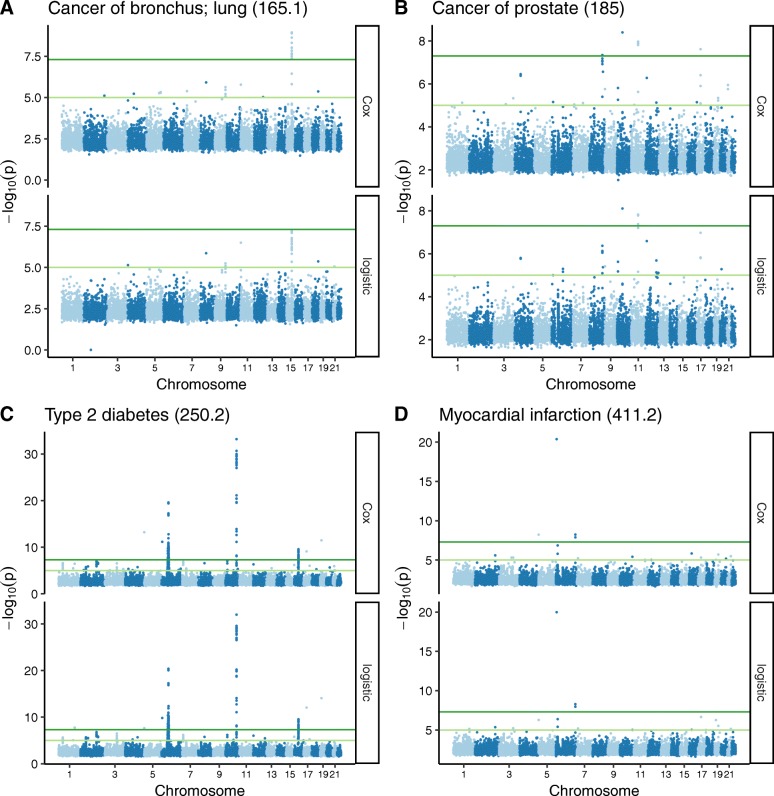
Manhattan plots of GWAS results using Cox and logistic regression for four phenotypes (phecode in parentheses). For each phenotype, only associations having mean(−log_10_(P)) ≥ 2 are shown. Dark green lines correspond to *P* = 5·10^− 8^ and light green lines correspond to *P* = 10^− 5^

We next compared the two methods using genetic data linked to electronic health records. We selected a cohort of 49,792 individuals of European ancestry, genotyped using the Illumina MEGA platform. We defined 50 phenotypes from the EHR, with the number of cases per phenotype ranging from 104 to 7972 (Additional file 1: Table S1). For each phenotype, we used Cox regression and logistic regression to run a GWAS on 795,850 common SNPs (including terms for principal components of genetic ancestry, Additional file 2: Fig. S1). Overall, the two methods gave similar results (Manhattan plots and QQ plots for four phenotypes in Fig. 2 and Additional file 2: Fig. S2). The *p*-values were highly correlated and the genomic inflation factors for both methods were generally slightly greater than 1 (Additional file 2: Fig. S3A-B). In addition, although coefficients from the two methods have different interpretations with different assumptions, the hazard ratios from Cox regression were strongly correlated with the odds ratios from logistic regression (R = 0.9997; Additional file 2: Fig. S3C). For associations with a mean -log_10_(P) ≥ 5, however, the *p*-value from Cox regression tended to be moderately lower than the p-value from logistic regression (Additional file 2: Fig. S3D-E). Cox regression also resulted in consistently smaller standard errors of coefficient estimates (Additional file 2: Fig. S3F). Across the 50 phenotypes, the total number of statistically significant associations was 7340 for Cox regression and 7109 for logistic regression (*P* ≤ 5·10^− 8^).

**Fig. 3:**
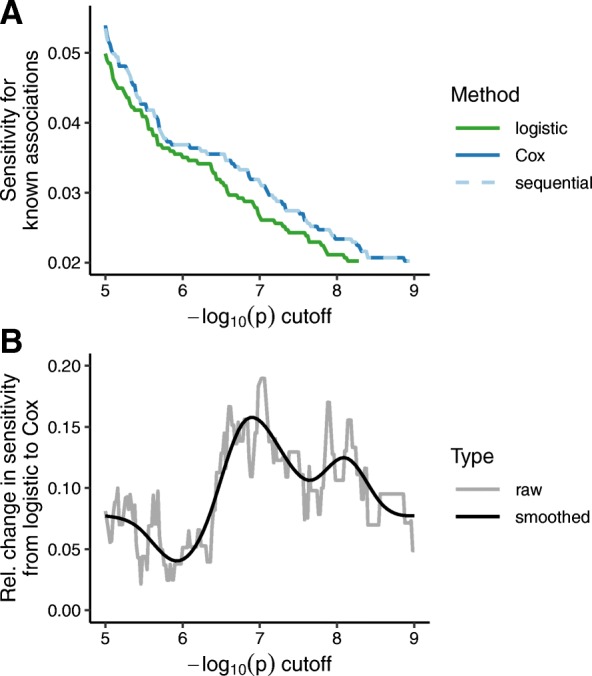
Comparing Cox regression and logistic regression for the ability to detect known genotype-phenotype associations for the 50 phenotypes analyzed. Known significant associations (*P* ≤ 5·10^− 8^) were curated from the NHGRI-EBI GWAS Catalog and aggregated by LD for each phenotype. **a** Sensitivity of each method, i.e., fraction of known and tested associations that gave a *p*-value less than or equal to the specified cutoff. The sequential strategy used the *p*-value from Cox regression, if the unadjusted *p*-value from logistic regression was ≤10^− 4^. The sequential line overlaps the Cox line. **b** Relative change in sensitivity between logistic and Cox regression, i.e., difference between the sensitivities for Cox and logistic, divided by the sensitivity for logistic. The gray line corresponds to the raw value at each cutoff, while the black line corresponds to the smoothed value according to a penalized cubic regression spline in a generalized additive model

We next used the GWAS results from the 50 phenotypes to evaluate each method’s ability to detect known associations from the NHGRI-EBI GWAS Catalog (Additional file 3: Table S2). Across a range of p-value cutoffs, Cox regression had approximately 10% higher relative sensitivity compared to logistic regression (Fig. 3). As in our simulations, the improvement in sensitivity was maintained by the sequential strategy of logistic followed by Cox.

**Fig. 4:**
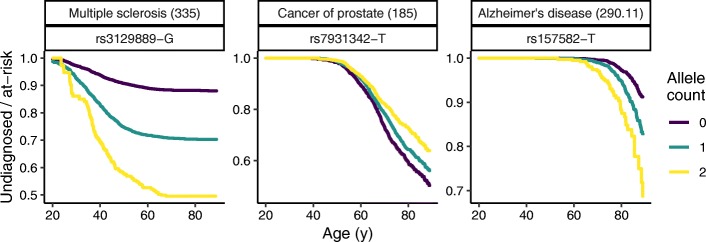
Kaplan-Meier curves for three phenotype-SNP pairs, showing the fraction of at-risk persons still undiagnosed as a function of age and allele count. For each phenotype, the corresponding phecode is in parentheses. As in the GWAS, diagnosis was defined as the second date on which a person received the given phecode. The curves do not account for sex or principal components of genetic ancestry, and thus are not exactly equivalent to the Cox regression used for the GWAS

In parallel to quantifying associations using Cox regression, it is natural to visualize them using Kaplan-Meier curves. For various phenotype-SNP pairs, we therefore plotted the number of undiagnosed individuals divided by the number at risk as a function of age and genotype (Fig. 4). These curves highlight not only a phenotype’s association with genotype, but also its characteristic age-dependent diagnosis rate.

## Discussion

The key piece of additional information required in Cox regression is the time to event. Thus, whereas an odds ratio from logistic regression represents the ratio of cumulative risk over all time, a hazard ratio from Cox regression represents the ratio of instantaneous risk at any given time (the strong correlation between the two quantities in our empirical data is likely due to low event rates and a valid proportional hazards assumption). In our analysis of EHR data, the time to event corresponded to the age at which a person either received a particular diagnosis code for the second time or was censored. Although acquisition of a diagnosis code is only an approximation for onset of a phenotype, the Kaplan-Meier curves for multiple phenotypes suggest that this approximation is valid [8–10].

To account for the fact that most individuals in our data are not observed from birth, we used the age of each individual’s first visit. This formulation of Cox regression, with left truncation and right censoring, corresponds to a counting process [11] and is not currently available in recently published software packages for GWAS of time-to-event outcomes [12, 13]. Furthermore, Cox regression is not available at all in popular GWAS tools such as PLINK. Thus, the implementation of Cox regression we used was not optimized for GWAS. Future work should make it possible to reduce the differences in computational cost and ease of use between Cox regression and logistic regression. In the meantime, we recommend the sequential strategy of logistic followed by Cox [7]. Although the initial threshold for logistic regression is arbitrary, our results suggest that a relatively loose threshold (e.g., *P* ≤ 10^− 4^) is likely to catch all significant associations without appreciably increasing computational cost.

Our use of the GWAS Catalog has multiple limitations. First, both methods showed low sensitivity, likely because for half of the 50 phenotypes, the number of EHR-derived cases was in the hundreds, whereas the number of cases from GWAS Catalog studies for these phenotypes was in the thousands. Thus, our analyses were underpowered for many SNP-phenotype associations. Second, the majority of studies in the GWAS Catalog followed a case-control design and quantified associations using either logistic or linear regression, not Cox regression. Thus, although the GWAS Catalog is the closest we have to a gold standard, it was important that our analyses of simulated data and empirical data gave consistent results.

## Conclusions

Here we used Cox regression to model the time to a single event, i.e., diagnosis of a particular phenotype. In the future, more sophisticated models may be able to account for subsequent response to treatment or semi-continuous traits such as lab values. We are especially interested in the potential of models that relax the proportional hazards assumption [14, 15] and the potential of Cox mixed models. The latter, like linear mixed models [16], use random effects to account for genetic relatedness, an increasingly important factor in EHR-linked samples [17]. Such an approach applied to large-scale datasets such as from the Million Veterans Program or the *All of Us* Research Program [18, 19], if appropriately adjusted for environmental and societal factors, may enable the creation of clinically useful polygenic hazard scores. Overall, as longitudinal, health-related data continue to grow, accounting for time through methods such as Cox regression may improve our ability to identify the genetic basis for human phenotypes.

## Methods

### Simulating linked genotype-phenotype data

We compared logistic regression and Cox regression in comprehensive simulations. As the effect sizes estimated by the two methods are not equivalent (i.e., odds ratio versus hazard ratio), we evaluated the methods in terms of average power and type I error calculated from true and false associations in each simulation.

The simulations and the analyses were designed to approximately mimic the empirical study on EHR data. In each simulation, we sampled minor allele counts for 800,000 SNPs in 50,000 individuals from a binomial distribution, with each minor allele’s probability independently simulated from the distribution of minor allele frequencies in the empirical genotype data. For simplicity, we simulated a haploid genome, i.e., each individual had only one allele at each SNP. Of the 800,000 minor alleles, 100 were declared as true risk alleles and the remaining 799,900 minor alleles were declared as false risk alleles by setting their coefficients to 0. We simulated data from both a Cox model and a logistic model. Due to computational burden, for each simulation model, we used 1000 simulations to assess true positive rates and 125 simulations to assess false positive rates.

To simulate data from a Cox model, the true event time was simulated from a multivariable Cox regression with baseline hazard generated from Exponential(λ) with λ = 10,000 and the parametric component including all SNPs. The coefficients of the 100 true alleles sampled from Unif(0.3, 0.5), i.e., a uniform distribution between 0.3 and 0.5, and coefficients of the remaining minor alleles were zeros. The censoring time was simulated from Gamma(1,1) and set at an upper bound of 2, which was designed to represent administrative censoring. The Gamma distribution is non-informative and allows non-uniform censoring [20]. The right censored observed event time was the minimum of the true event time and the censoring time. The left truncation time was simulated from Unif(0, 0.1). Individuals whose censoring time or event time was less than the truncation time were removed from the dataset (mean 9% of individuals, range 6.61 to 9.48%). The mean event rate was 30.2% (range 6.66 to 66.9%). For each SNP in each simulation, we ran univariate Cox regression (with left truncation) and multivariable logistic regression. The latter included two additional variables: age at event and difference between age at truncation and age at event, both encoded as restricted cubic splines with five knots.

To simulate data from a logistic model, age (a surrogate of the true event time) was simulated from a normal distribution with mean 60 and standard deviation 5. The event indicator was simulated from a logistic regression model with all SNPs and age. The coefficients were sampled from Unif(0.3, 0.7) for the 100 true alleles, zero for the remaining null minor alleles, and 0.001 for age. The censoring time was simulated from Unif(50, 85) [21], leading to 31.8% mean event rate (range 6.48 to 68.3%). For each SNP in each simulation, we ran univariate Cox regression (without truncation, since no truncation time was simulated) and multivariable logistic regression. The latter included an additional variable for age at event, which was encoded as a restricted cubic splines with five knots.

Statistical significance was based on Bonferroni correction with an overall type I error rate of 0.01, 0.05, and 0.1.

### Processing the empirical genotype data

Our empirical data came from the Vanderbilt Synthetic Derivative (a database of de-identified electronic health records) and BioVU (a DNA biobank linked to the Synthetic Derivative) [22]. We used a cohort that was genotyped using the Illumina MEGA platform. To identify individuals of European ancestry (the majority in BioVU), we used STRUCTURE to create three clusters, keeping those individuals who had a score ≥ 0.9 for the cluster that corresponded to European ancestry [23]. We then filtered SNPs to keep those that had a minor allele frequency ≥ 0.01, call rate ≥ 0.95, *p*-value of Hardy-Weinberg equilibrium ≥0.001, and p-value of association with batch ≥10^− 5^. To calculate the principal components (PCs) of genetic ancestry, we followed the recommended procedure of the SNPRelate R package v1.16.0 [24]. Specifically, we pruned SNPs based on a linkage disequilibrium (LD) threshold r = 0.2, then used the randomized algorithm to calculate the first 10 PCs [25].

### Identifying phenotypes for empirical study

To compare the ability of Cox and logistic regression to detect known associations, we selected 50 phenotypes that could be studied with EHR data and which also had known associations from the NHGRI-EBI GWAS Catalog v1.0.2 r2018-08-30 (Additional file 1: Table S1) [26]. The phenotypes were selected before the analysis was performed. We only considered GWAS Catalog studies with at least 1000 cases and 1000 controls of European ancestry (Additional file 3: Table S2). We manually mapped studies and their corresponding traits to EHR phenotypes using phecodes, which are derived from billing codes [27]. For each phenotype, we defined cases as individuals who received the corresponding phecode on two distinct dates, and controls as individuals who have never received the corresponding phecode. Each phenotype had at least 100 cases.

### Running the GWAS on empirical data

For both Cox regression and logistic regression, the linear model included terms for genotype (assuming an additive effect) and the first four principal components of genetic ancestry (Additional file 2: Fig. S1). Depending on the phenotype, the model either included a term for biological sex or the cases and controls were limited to only females or only males. For logistic regression, the model also included terms for age at the time of last visit (modeled as a cubic smoothing spline with three degrees of freedom) and the length of time between first visit and last visit. For Cox regression, the model used the counting process formulation, such that time 1 (left truncation time) corresponded to age at first visit ever and time 2 (event time or right censoring time) corresponded to age on the second distinct date of receiving the given phecode (for cases) or age at last visit (for controls).

Logistic regression was run using PLINK v2.00a2LM 64-bit Intel (30 Aug 2018) [28]. Cox regression was run in R v3.5.1 using the agreg.fit function of the survival package v2.43–3. The agreg.fit function is normally called internally by the coxph function, but calling agreg.fit directly is faster. The total runtimes for the GWASes of the 50 phenotypes using logistic and Cox regression (parallelized on 36 cores) were 1.6 days and 7.1 days, respectively.

### Comparing the GWAS results to the GWAS catalog

For each mapped study from the GWAS Catalog, we only considered SNPs having an association *P* ≤ 5·10^− 8^. For each phenotype, we then used LDlink [29] to group the associated SNPs into LD blocks (r^2^ ≥ 0.8). For each associated SNP for each phenotype, we then determined which SNPs on the MEGA platform were in LD with that SNP (r^2^ ≥ 0.8), and assigned those SNPs to the corresponding phenotype and LD block. Using the EHR-based GWAS results, we then calculated the sensitivity of Cox regression and logistic regression based on the number of phenotype-LD block pairs for which at least one SNP in that LD block had a *p*-value less than a given p-value cutoff (across a range of cutoffs).

## Supplementary information

**Additional file 1: Table S1.** Information for each of the 50 phenotypes.

**Additional file 2: Figs. S1-S3.** Supplemental figures for principal components of genetic ancestry and GWAS results using Cox and logistic regression.

**Additional file 3: Table S2.** Mapping between phecodes and GWAS Catalog study accessions.

## Data Availability

Access to individual-level EHR and genotype data is restricted by the IRB. Code and summary-level results are available at 10.6084/m9.figshare.7881146.

## Abbreviations

EHR: electronic health record
GWAS: genome-wide association study
LD: linkage disequilibrium
PC: principal component
PheWAS: phenome-wide association study
SNP: single-nucleotide polymorphism

## References

[1] Denny JC, Van Driest SL, Wei W-Q, Roden DM (2018). The Influence of Big (Clinical) Data and Genomics on Precision Medicine and Drug Development. Clin Pharmacol Ther..

[2] Aschard H, Vilhjálmsson BJ, Greliche N, Morange P-E, Trégouët D-A, Kraft P (2014). Maximizing the power of principal-component analysis of correlated phenotypes in genome-wide association studies. Am J Hum Genet..

[3] Cortes A, Dendrou CA, Motyer A, Jostins L, Vukcevic D, Dilthey A (2017). Bayesian analysis of genetic association across tree-structured routine healthcare data in the UK Biobank. Nat Genet..

[4] Collett D. Modelling Survival Data in Medical Research: CRC Press; 2015.

[5] Cox DR (1972). Regression models and life-tables. J R Stat Soc Series B Stat Methodol..

[6] van der Net JB, Janssens ACJW, Eijkemans MJC, Kastelein JJP, Sijbrands EJG, Steyerberg EW (2008). Cox proportional hazards models have more statistical power than logistic regression models in cross-sectional genetic association studies. Eur J Hum Genet..

[7] Staley JR, Jones E, Kaptoge S, Butterworth AS, Sweeting MJ, Wood AM (2017). A comparison of Cox and logistic regression for use in genome-wide association studies of cohort and case-cohort design. Eur J Hum Genet..

[8] Hebert LE, Scherr PA, Beckett LA, Albert MS, Pilgrim DM, Chown MJ (1995). Age-specific incidence of Alzheimer’s disease in a community population. JAMA..

[9] Cottrell DA, Kremenchutzky M, Rice GP, Koopman WJ, Hader W, Baskerville J (1999). The natural history of multiple sclerosis: a geographically based study. 5. The clinical features and natural history of primary progressive multiple sclerosis. Brain..

[10] Grönberg H (2003). Prostate cancer epidemiology. Lancet..

[11] Andersen PK, Gill RD (1982). Cox’s Regression Model for Counting Processes: A Large Sample Study. Ann Stat..

[12] Syed H, Jorgensen AL, Morris AP (2017). SurvivalGWAS_SV: software for the analysis of genome-wide association studies of imputed genotypes with “time-to-event” outcomes. BMC Bioinformatics..

[13] Rizvi AA, Karaesmen E, Morgan M, Preus L, Wang J, Sovic M (2018). gwasurvivr : an R package for genome wide survival analysis. Bioinformatics.

[14] Hess KR (1994). Assessing time-by-covariate interactions in proportional hazards regression models using cubic spline functions. Stat Med..

[15] Schemper M, Wakounig S, Heinze G (2009). The estimation of average hazard ratios by weighted Cox regression. Stat Med..

[16] Yang J, Zaitlen NA, Goddard ME, Visscher PM, Price AL (2014). Advantages and pitfalls in the application of mixed-model association methods. Nat Genet..

[17] Staples J, Maxwell EK, Gosalia N, Gonzaga-Jauregui C, Snyder C, Hawes A (2018). Profiling and Leveraging Relatedness in a Precision Medicine Cohort of 92,455 Exomes. Am J Hum Genet..

[18] Gaziano JM, Concato J, Brophy M, Fiore L, Pyarajan S, Breeling J (2016). Million Veteran Program: A mega-biobank to study genetic influences on health and disease. J Clin Epidemiol..

[19] Denny JC, Rutter JL, Goldstein DB, Philippakis A, Smoller JW, All of Us Research Program Investigators (2019). The “All of Us” Research Program. N Engl J Med.

[20] Li C (2018). Two-sample tests for survival data from observational studies. Lifetime Data Anal..

[21] Hong HG, Kang J, Li Y (2018). Conditional screening for ultra-high dimensional covariates with survival outcomes. Lifetime Data Anal..

[22] Danciu I, Cowan JD, Basford M, Wang X, Saip A, Osgood S (2014). Secondary use of clinical data: the Vanderbilt approach. J Biomed Inform..

[23] Pritchard JK, Stephens M, Donnelly P (2000). Inference of population structure using multilocus genotype data. Genetics..

[24] Zheng X, Levine D, Shen J, Gogarten SM, Laurie C, Weir BS (2012). A high-performance computing toolset for relatedness and principal component analysis of SNP data. Bioinformatics..

[25] Galinsky KJ, Bhatia G, Loh P-R, Georgiev S, Mukherjee S, Patterson NJ (2016). Fast Principal-Component Analysis Reveals Convergent Evolution of ADH1B in Europe and East Asia. Am J Hum Genet..

[26] Buniello A, MacArthur JAL, Cerezo M, Harris LW, Hayhurst J, Malangone C (2019). The NHGRI-EBI GWAS Catalog of published genome-wide association studies, targeted arrays and summary statistics 2019. Nucleic Acids Res..

[27] Denny JC, Bastarache L, Ritchie MD, Carroll RJ, Zink R, Mosley JD (2013). Systematic comparison of phenome-wide association study of electronic medical record data and genome-wide association study data. Nat Biotechnol..

[28] Chang CC, Chow CC, Tellier LC, Vattikuti S, Purcell SM, Lee JJ (2015). Second-generation PLINK: rising to the challenge of larger and richer datasets. Gigascience..

[29] Machiela MJ, Chanock SJ (2015). LDlink: a web-based application for exploring population-specific haplotype structure and linking correlated alleles of possible functional variants. Bioinformatics..

