# Supplementary material for: Cox regression increases power to detect genotype-phenotype associations in genomic studies using the electronic health record
Source: BMC Genomics. 2019 Nov 4;20:805. doi: 10.1186/s12864-019-6192-1 (PMC6829851; doi:10.1186/s12864-019-6192-1)
Supplement: Supplementary file 2 — Additional file 2: Figs. S1-S3. Supplemental figures for principal components of genetic ancestry and GWAS results using Cox and logistic regression. [file 12864_2019_6192_MOESM2_ESM.pdf]

Supplemental Figures for:

Cox regression increases power to detect genotype-phenotype associations in genomic studies using the electronic health record

Jacob J. Hughey[1,2,*], Seth D. Rhoades[1], Darwin Y. Fu[1], Lisa Bastarache[1], Joshua C. Denny[1,3], and Qingxia Chen[1,4]

[1]Department of Biomedical Informatics, Vanderbilt University Medical Center, Nashville, TN
[2]Department of Biological Sciences, Vanderbilt University, Nashville, TN
[3]Department of Medicine, Vanderbilt University Medical Center, Nashville, TN
[4]Department of Biostatistics, Vanderbilt University Medical Center, Nashville, TN

*To whom all correspondence should be addressed: jakejhughey@gmail.com

Figure S1

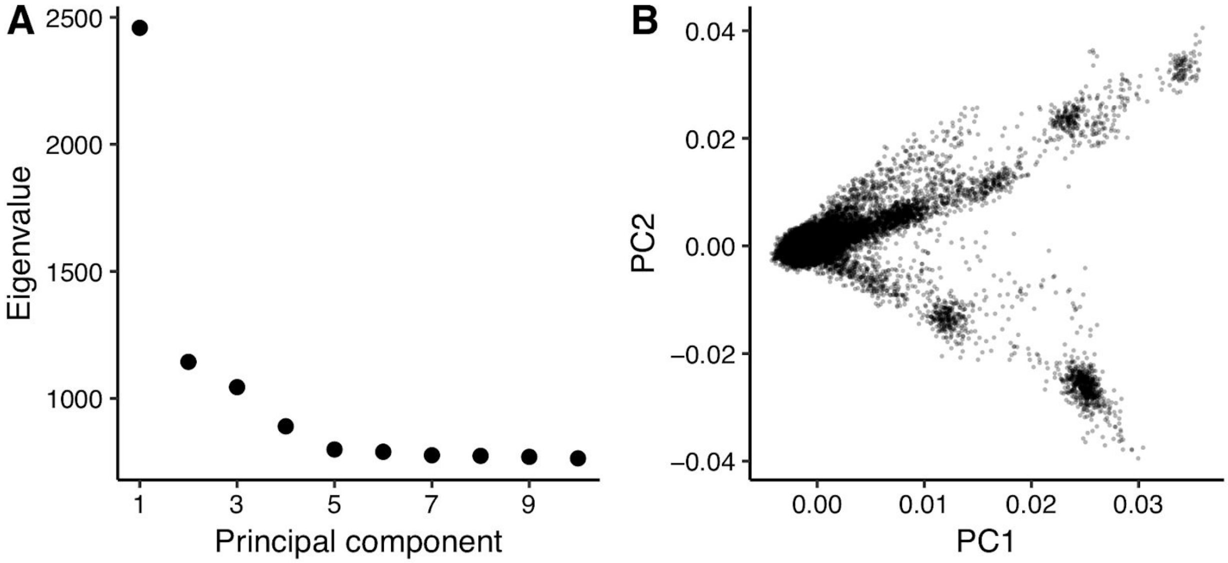

Principal components of genetic ancestry, calculated using SNPRelate. **(A)** Eigenvalues for the top 10 principal components. **(B)** Scatterplot of PC2 vs. PC1, where each point corresponds to a subject in the cohort.

Figure S2

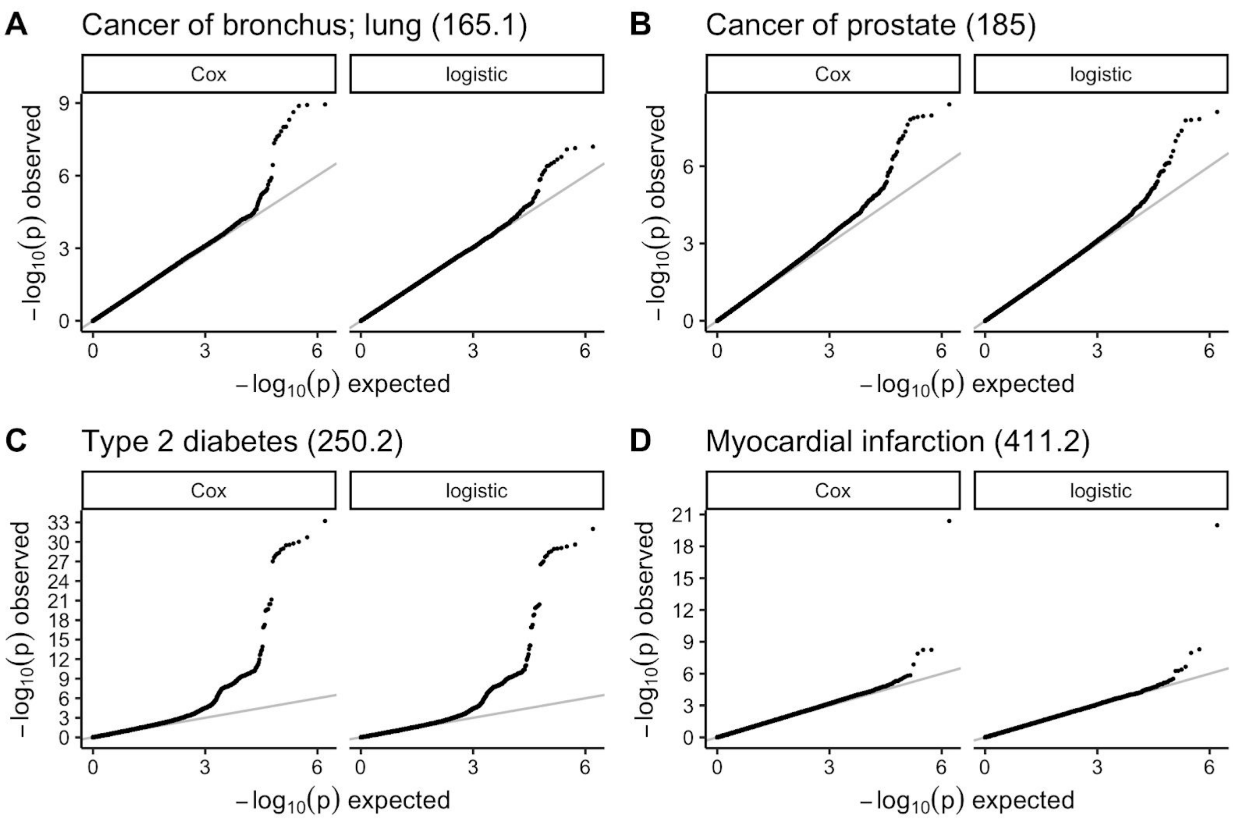

QQ-plots of GWAS results using Cox and logistic regression for four phenotypes (phecode in parentheses). Each point corresponds to a SNP. The gray line indicates y = x.

Figure S3

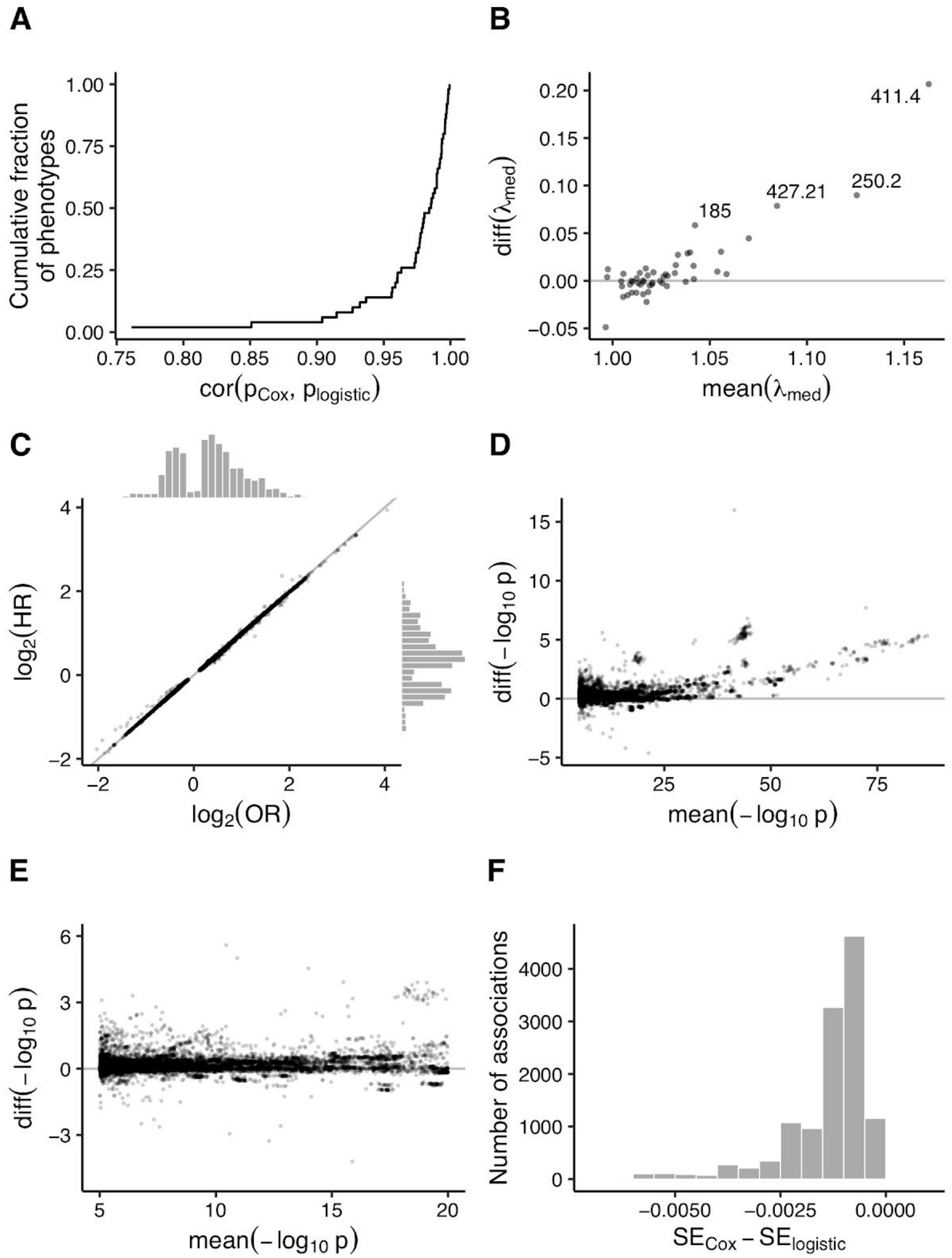

Comparing the results of GWASes based on Cox regression and logistic regression for 50 phenotypes. In all mean-difference plots, a difference > 0 indicates a higher value for Cox than for logistic. **(A)** Empirical cumulative distribution function of the Pearson correlation between p-values for each phenotype. **(B)** Mean-difference plot of the genomic inflation factor. Each point corresponds to a phenotype. Phenotypes with a difference > 0.05 are labeled with the

corresponding phecode (411.4: coronary atherosclerosis, 250.2: type 2 diabetes, 427.21: atrial fibrillation, 185: prostate cancer). **(C)** Scatterplot and marginal histograms of $\log_2$(effect size) for Cox and logistic regression, calculated as $log_2(e^{\beta})$, where β is the coefficient for genotype. HR refers to hazard ratio from Cox regression, OR refers to odds ratio from logistic regression. In panels C-E, each point corresponds to a SNP-phenotype pair. Panels C-F include only those associations for which $P \leq 10^{-5}$ for either Cox or logistic regression. **(D)** and **(E)** Mean-difference plots, between Cox and logistic regression, of $-\log_{10}(P)$. The two plots differ in their axis limits. Each point corresponds to a SNP-phenotype pair. **(F)** Histogram of the difference in standard error of the coefficient estimate.
